# Mollaret’s Syndrome: A Case Report

**DOI:** 10.7759/cureus.38559

**Published:** 2023-05-04

**Authors:** Vivien M Edi, Prasad Rao, Joanna O Igo, Amaka S Odega, Elizabeth Soladoye, Chidalu N Ibeneme, Okelue E Okobi

**Affiliations:** 1 Internal Medicine, Wellstar Kennestone Hospital, Marietta, USA; 2 Family Medicine, West Ottawa Specialty Care, Ottawa, CAN; 3 Community and Family Medicine, Windsor University School of Medicine, Cayon, KNA; 4 Public Health, University of South Wales, Cardiff, GBR; 5 Family Medicine, Olabisi Onabanjo University, Ago Iwoye, NGA; 6 Psychiatry, PsycIME, London, CAN; 7 Internal Medicine, Piedmont Athens Regional, Athens, USA; 8 Family Medicine, Abia State University, Uturu, NGA; 9 Family Medicine, Medficient Health LLC, Laurel, USA; 10 Family Medicine, Lakeside Medical Center, Belle Glade, USA

**Keywords:** rare disease, viral meningitis, mollaret's lymphocytic meningitis, mollaret's syndrome, mollaret's meningitis

## Abstract

Benign recurrent aseptic meningitis is a rare condition characterized by recurring, self-limited episodes of aseptic meningitis. Meningeal irritation typically occurs first, accompanied by fever and mononuclear cell pleocytosis. The diagnosis is only made after other known causes of lymphocytic meningitis have been excluded. Resolution typically occurs within two to seven days without residual neurological deficit. Aseptic meningitis is most frequently caused by viruses; Mollaret’s meningitis has been linked to the herpes simplex virus 2 (HSV 2). It is unclear if prophylactic medication is indicated for these patients. We describe a patient who was experiencing her seventh episode of aseptic meningitis.

## Introduction

Mollaret's syndrome, a rare presentation that has been reported in a few scientific journals, is a term that can refer to two distinct medical conditions: Mollaret's meningitis and Mollaret's lymphocytic meningitis. Note that while both conditions share similarities, they are distinct and should not be confused with each other. Mollaret's meningitis is characterized by recurrent episodes of meningismus and typically goes away on its own within a week or so. On the other hand, Mollaret's lymphocytic meningitis is a recurrent or chronic inflammation of the meninges that can cause long-term neurological damage [1-4].

Mollaret’s meningitis is a rare disease characterized by recurrent episodes of aseptic lymphocytic meningitis that last for a few days and are followed by symptom-free intervals. The disease was first described by the French neurologist Pierre Mollaret in 1944 [4]. The episodes of meningitis are usually caused by herpes simplex virus type 2 (HSV-2) infection, although other viral and non-viral causes have also been reported. The disease is usually benign and does not cause long-term sequelae. While Mollaret's lymphocytic meningitis is a type of recurrent aseptic meningitis that is similar to Mollaret's meningitis, its underlying cause and pathophysiology are less well-defined. A recent report by Willmann et al. hypothesizes that a deficiency of toll-like receptor 3, involved in the innate immune system response to viral infection, may predispose to this recurrent lymphocytic meningitis, but it is unclear whether this proposed pathophysiology can be extrapolated to reports of Mollaret's meningitis [2].

The symptoms of Mollaret's syndrome typically include fever, headache, stiff neck, nausea, vomiting, and photophobia, as well as various neurological symptoms. However, the symptoms may vary between episodes and may not always be present. The episodes are usually brief, lasting between two to five days, and are followed by symptom-free intervals [1-4]. In addition to these common symptoms, Mollaret's syndrome can also present with a variety of neurological symptoms [5-14]. These may include confusion, altered consciousness, seizures, paresthesia, and cranial nerve palsies [1-4]. Other less common symptoms that have been reported in association with Mollaret's syndrome include myalgias, arthralgias, rash, and diarrhea [1-2]. It is important to note that the symptoms of Mollaret's syndrome may vary between episodes and may not always be present. In some cases, the episodes may be asymptomatic or may only present with mild symptoms [1-4].

## Case presentation

A 27-year-old white female immigrant presented with a constellation of symptoms including malaise, headache, fever, and neck pain, which started a day prior to presentation. She has a significant medical history dating back to 2014 when she first presented to our center with similar symptoms and was diagnosed with, and managed for lymphocytic meningitis. Since then, she has presented about seven times with similar symptoms. 

During the hospitalizations for those episodes, various tests were conducted, including HSV polymerase chain reaction (PCR), HIV antibody, enteroviral PCR, QuantiFERON-TB Gold (QIAGEN N.V., Hilden, Germany), rapid plasma reagin (RPR), and brain magnetic resonance imaging (MRI), which were all negative except for HSV PCR. A summary of lumbar punctures since 2014 revealed pleocytosis with a leukocyte count ranging from 50-260 leukocytes per cubic millimeter, with elevations in both mononuclear and polynuclear cells. Despite repeated cerebrospinal fluid (CSF) cultures, no causative bacterial organism was identified. PCR analysis over the years revealed the presence of HSV-2 in some of her admissions (2014, 2015, 2019, 2021, and the current admission) (Table 1). 

**Table 1:** Viral culture and PCR Over the years, viral culture and PCR has yielded mixed result for HSV-2. At the current presentation, CSF culture was negative; CSF PCR was positive for HSV-2. PCR: polymerase chain reaction; HSV: herpes simplex virus

Component	9 years ago (July 26, 2014)	9 years ago (October 29, 2014)	8 years ago (March 26, 2015)	4 years ago (August 9, 2019)	2 years ago (May 8, 2021)	Current presentation (January 3, 2023)
Viral source	Cerebrospinal fluid	Venous blood	Cerebrospinal fluid	Cerebrospinal fluid	Cerebrospinal fluid	Cerebrospinal fluid
HSV 1 DNA	Not detected	Not detected	Not detected	Not detected	Not detected	Not detected
HSV 2 DNA	Detected	Not detected	Detected	Detected	Detected	Detected

In cases where HSV was confirmed by PCR, antiviral therapy (acyclovir was usually given at a dose of 5-10 mg/kg) in conjunction with other therapy for aseptic meningitis (dexamethasone, electrolyte, and fluid support as needed). Following discharge from those hospitalizations, she was usually prescribed valacyclovir 500 mg daily for prophylaxis but reported noncompliance due to undisclosed personal reasons.

The patient also reported that prior to visiting our center in 2014, she had a positive medical history of similar symptoms and recurrent meningitis; although we were unable to retrieve the out-of-country medical records of her previous diagnoses prior to 2014, neither could she remember at what year exactly her symptoms started. In addition to her history of recurrent meningitis, she also reported a history of recurrent genital herpes, hidradenitis suppurativa, and about three previous spontaneous abortions prior to 2014 and she is unsure if any workup was done. She had no other significant surgical or medical history. Medication history, family history, and social history were all negative for any concerns. 

During the patient examination, she exhibited signs of photophobia and neck stiffness, despite being oriented to time, place, and person. Further neurological examination did not reveal any focal abnormalities. Vital signs, such as blood pressure, pulse, temperature, and respirations, were within normal ranges, with blood pressure measuring 118/73 mm Hg, pulse measuring 72 beats per minute, temperature measuring 100.2^o^F (37.9^o^C), and respirations measuring 16 breaths per minute. Additionally, on further laboratory workup, the patient presented with mild anemia (hemoglobin of 11.6) and normal neutrophil counts. Other laboratory work including urinalysis was within normal ranges. A computerized tomography (CT) scan of the head was conducted and did not reveal any acute intracranial abnormalities. However, further evaluation with lumber puncture (LP) and CSF analysis showed elevated white blood cells, primarily neutrophils, though cultures were negative (Table 2). 

**Table 2:** CSF findings from the patient’s first and latest admission

CSF (4^th^ Vial)	WBC (/mm^3^)	Lymphocytes (%)	Glucose (mg/dL)	Protein (mg/dL)
First Admission (2014)	67	51	52	31
Lastest Admission (2023)	216	93	47	113
Normal Value	0-5	60–70	40-70	<40 mg/dL

**Figure 1:**
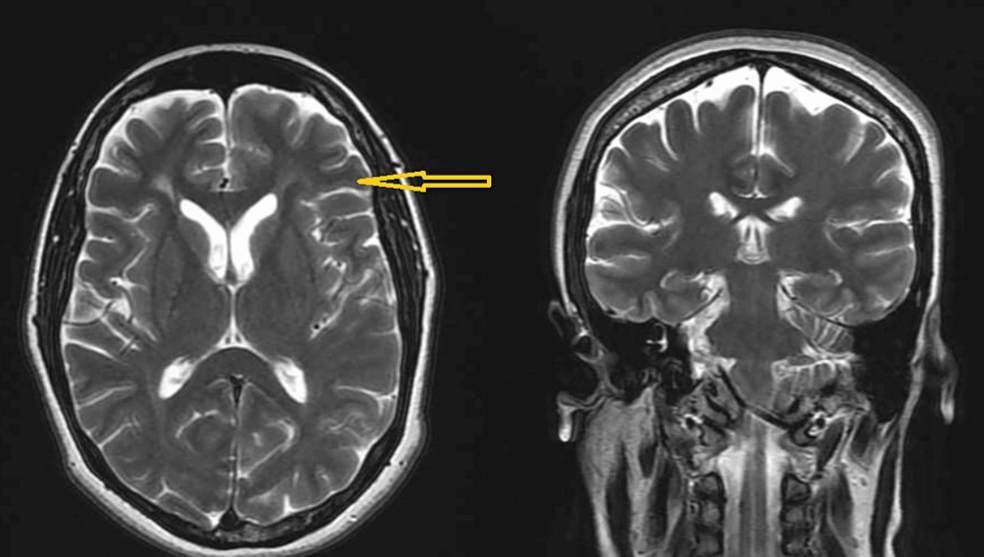
Axial and coronal T2W brain MR images showing normal findings

Other workups at this visit included a brain MRI (Figure 1) that depicted well-defined and distinct brain structures demarcated by clear boundaries. Furthermore, it revealed normal bilateral symmetry between the brain's hemispheres without any discernible lesions or abnormalities. The cortex demonstrated uniform and even contours. The differentiation between the white and gray matter was unremarkable. The brainstem exhibited regular dimensions and shape without any evidence of compression or anomalous growths.

Empiric treatment with intra-venous antibiotics (including vancomycin) and acyclovir was initiated on day 1, and a team of specialists from multiple disciplines was involved in exploring potential alternative diagnoses such as venous malformation. However, it was ultimately determined that these were unlikely. 

Despite remaining febrile, the patient's hemodynamic status remained stable, and empiric antibiotics were discontinued on the second day of hospitalization after a negative CSF culture. However, CSF PCR ultimately confirmed the presence of HSV-2, leading to an increase in the acyclovir dose to 10 mg/kg every eight hours and dexamethasone 4 mg every eight hours. By the fourth day of hospitalization, the patient's clinical status had improved, and fevers had subsided. By the fifth day, the patient was discharged on valacyclovir for chronic suppression therapy.

## Discussion

The clinical presentation of the patient, the cytomorphologic features of the CSF, and imaging findings are consistent with a diagnosis of Mollaret’s syndrome (possibly Mollarets lymphocytic meningitis). The exact cause of this rare neurological disorder characterized by recurrent episodes of meningitis and inflammation of the membranes surrounding the brain and spinal cord is poorly understood. Still, several factors have been implicated. Viral infections, autoimmune disorders, genetic factors, and environmental factors may all play a role in developing this rare neurological disorder. The patient’s CSF viral culture over the years during her episodes of illness also showed this pattern. As shown in the result panel, HSV was cultured in some episodes and, in other episodes, was negative. These recurrent episodes of viral and non-viral cultures have also been documented in other scientific manuscripts with an attempt to associate them with this rare syndrome [1-4,7]

A study suggested that viral infections may potentially cause Mollaret’s syndrome. In particular, HSV and varicella-zoster virus (VZV) have been implicated in causing recurrent meningitis episodes in some individuals [1]. Several other studies have suggested that Mollaret’s syndrome may be caused by a viral infection [1-4,6-14]. In particular, HSV and VZV have been implicated. HSV is a common virus that can cause cold sores or genital herpes, while VZV is the virus that causes chickenpox and shingles. Studies have shown that these viruses can remain dormant in the body and reactivate to cause recurrent meningitis episodes in some people [1-4].

Autoimmune disorders, in which the immune system attacks the body’s tissues, have also been linked to Mollaret’s syndrome. In the index case, it was unclear if an autoimmune response drove the patient’s condition. Some authors have also suggested this immunologic etiopathogenesis. For example, studies have shown that antiphospholipid antibodies, associated with autoimmune disorders like systemic lupus erythematosus (SLE), may present in some people with Mollaret’s syndrome. Additionally, autoimmune disorders such as SLE and Behcet’s disease have also been associated with recurrent meningitis [1-4]

Genetic factors may also play a role in developing Mollaret’s syndrome. There is a possibility of a connection between Mollaret's meningitis and familial Mediterranean fever (FMF), which is an inherited autoinflammatory disorder [15]. While reports have linked the two conditions in a small number of patients, there is currently no data to establish a definitive causal relationship between them. Also, a genetic mutation in the *TLR3, UNC-93B * gene has also been identified in some individuals with Mollaret’s syndrome [2]. This mutation is involved in regulating inflammation and may lead to an overactive immune response and increased inflammation in the brain and spinal cord, leading to recurrent meningitis episodes [1-4]. Studies have identified a genetic mutation in the *TNFAIP3* gene, which regulates inflammation in some people with Mollaret’s syndrome. This mutation may lead to an overactive immune response and increased brain and spinal cord inflammation, leading to recurrent meningitis episodes.

Diagnosing Mollaret’s syndrome, also known as recurrent benign lymphocytic meningitis, can be challenging, as no specific tests are available for this rare neurological disorder. Instead, the diagnosis is typically based on a combination of clinical symptoms, laboratory tests, and imaging studies. This patient was worked up along these lines, with negative radiological imaging results and blood and CSF cultures negative for bacteria.

One of the critical laboratory tests used to diagnose Mollaret’s syndrome is CSF analysis. Some researchers found that individuals with Mollaret’s syndrome typically have elevated levels of lymphocytes in their CSF and protein levels [1-4,7]. Other laboratory findings in Mollaret’s syndrome may include high CSF IgG and IgA antibody levels, indicating an autoimmune response [1-4].

As seen in this case presentation, viral testing may also be used to help diagnose Mollaret’s syndrome in addition to CSF analysis. PCR testing can detect the presence of viral DNA in the CSF, which can help identify the causative agent for recurrent meningitis episodes. In particular, HSV and VZV are often implicated in Mollaret’s syndrome [1-4].

Imaging studies such as MRI may also be used to help diagnose Mollaret’s syndrome. For example, MRI can help identify brain or spinal cord structural abnormalities contributing to recurrent meningitis episodes. In some cases, MRI may also reveal evidence of brain and spinal cord inflammation, which can support a diagnosis of Mollaret’s syndrome [1-4].

Treating Mollaret’s syndrome mainly involves managing symptoms during acute episodes and preventing future attacks. Overall, the treatment of Mollaret’s syndrome is tailored to the individual case and should be determined in consultation with a healthcare professional. In the current case, the practical choice of antibacterial and antiviral was initiated at the index presentation because of the previous patterns of atypical CSF findings. Some authors have suggested that Mollaret’s meningitis may be managed with symptomatic care [7, 13].The illness resolves completely without any neurological sequelae. The article further suggests that for patients with a history of noninfectious Mollaret’s meningitis who present with meningismus, aseptic meningitis can be confirmed with a lumbar puncture and treated accordingly. Furthermore, treatment options for Mollaret’s syndrome vary depending on the case. Antiviral therapy has shown success in treating and preventing recurrences of Mollaret’s syndrome in some patients [13]. However, supportive care without prophylaxis has also been used in other cases with positive outcomes [11]. In cases where antiviral prophylaxis has failed, and there are repeated recurrences of similar symptoms, as described by some authors, further evaluation and consideration of alternative treatment options may be necessary [12,14]. It is debatable if our patient benefited from long-term prophylaxis.

There is limited information available regarding the prognosis of Mollaret’s syndrome. However, it is generally considered to have a good prognosis, as patients usually recover entirely between episodes without any lasting neurological deficits. Recurrent episodes of meningitis can occur at varying intervals, ranging from days to years [1-4]. In one case report, a patient with Mollaret’s syndrome experienced three episodes of meningitis over four years but had no residual neurological deficits after each episode and responded well to treatment with acyclovir [3]. Another case report described a patient who had two previous outbreaks of viral meningitis and presented with Mollaret’s syndrome. The patient had meningism without focal neurological deficits and recovered completely after treatment with acyclovir and steroids [2].

There is no specific cure for Mollaret’s syndrome, and treatment focuses on symptomatic relief and prevention of future attacks. The use of antiviral agents, such as acyclovir, has been suggested in some cases, but there is no conclusive evidence of their efficacy [11]. Nonsteroidal anti-inflammatory drugs (NSAIDs) are commonly used for their anti-inflammatory and analgesic effects in a variety of conditions, including meningitis caused by other pathogens such as bacterial and viral infections. However, while colchicine and NSAIDs may have potential therapeutic effects in the treatment of Mollaret's meningitis, there is limited evidence specifically addressing their use in this condition. A case report of a 32-year-old man with Mollaret's meningitis found that colchicine did not decrease the severity or frequency of attacks when administered prophylactically [12], while another case report suggests that prophylactic treatment with low-dose colchicine or nonsteroidal anti-inflammatory drugs (NSAIDs) reduces the frequency and severity of attacks in some patients [15]. Furthermore, a multicenter randomized controlled trial by Aurelius et al., RCT, Using initial treatment with 1g valacyclovir for three days and prophylactic 0.5 g valacyclovir or placebo twice daily for one year, concluded that there was a higher frequency of rebound meningitis, perhaps because the dose was too small to prevent the activation of HSV-2 in the CNS [14]. They further stated that valacyclovir should not be recommended for prophylaxis. This double-blinded trial that looked at patients with Mollaret’s meningitis who received suppressive therapy with 0.5 g valacyclovir twice daily concluded that prophylaxis failed to prevent the recurrence of meningitis but did reduce outbreaks of genital herpes [14].

## Conclusions

The treatment of Mollaret's syndrome is mainly supportive and aims to manage symptoms during acute episodes and prevent future attacks. Although no specific cure exists, prophylactic treatment with colchicine or NSAIDs may be helpful in reducing the frequency and severity of attacks. Anecdotal experiences and case reports are the primary sources of information regarding the effectiveness of antiviral therapy as prophylaxis for this condition. Whether increasing the dose of valacyclovir for better CNS penetration will affect the outcome remains uncertain. While it is suggested that patients with Mollaret’s meningitis may benefit from long-term prophylaxis, the ideal regime and duration of treatment have yet to be determined and appear to vary on a case-by-case basis. Further research is needed to determine the most effective treatment strategies for Mollaret's syndrome.
